# Adaptive Automation Triggered by EEG-Based Mental Workload Index: A Passive Brain-Computer Interface Application in Realistic Air Traffic Control Environment

**DOI:** 10.3389/fnhum.2016.00539

**Published:** 2016-10-26

**Authors:** Pietro Aricò, Gianluca Borghini, Gianluca Di Flumeri, Alfredo Colosimo, Stefano Bonelli, Alessia Golfetti, Simone Pozzi, Jean-Paul Imbert, Géraud Granger, Raïlane Benhacene, Fabio Babiloni

**Affiliations:** ^1^Department of Molecular Medicine, Sapienza University of RomeRome, Italy; ^2^BrainSigns Co. Ltd, Spin-off Company from Sapienza University of RomeRome, Italy; ^3^Neuroelectrical Imaging and BCI Lab, Fondazione Santa Lucia (IRCCS)Rome, Italy; ^4^Department of Anatomical, Histological, Forensic Medicine and Orthopedic Sciences, Sapienza University of RomeRome, Italy; ^5^DeepBlue srlRome, Italy; ^6^École Nationale de l'Aviation CivileToulouse, France

**Keywords:** passive brain-computer interface (pBCI), Adaptive Automation (AA), Air Traffic Management (ATM), electroencephalogram (EEG), mental workload, human factors, machine learning, human machine interaction

## Abstract

*Adaptive Automation* (AA) is a promising approach to keep the task workload demand within appropriate levels in order to avoid both the *under*- and *over-load* conditions, hence enhancing the overall performance and safety of the human-machine system. The main issue on the use of AA is how to trigger the AA solutions without affecting the operative task. In this regard, *passive Brain-Computer Interface* (pBCI) systems are a good candidate to activate automation, since they are able to gather information about the covert behavior (e.g., mental workload) of a subject by analyzing its neurophysiological signals (i.e., brain activity), and without interfering with the ongoing operational activity. We proposed a pBCI system able to trigger AA solutions integrated in a realistic *Air Traffic Management* (ATM) research simulator developed and hosted at ENAC (É*cole Nationale de l'Aviation Civile* of Toulouse, France). Twelve *Air Traffic Controller* (ATCO) students have been involved in the experiment and they have been asked to perform ATM scenarios with and without the support of the AA solutions. Results demonstrated the effectiveness of the proposed pBCI system, since it enabled the AA mostly during the high-demanding conditions (i.e., overload situations) inducing a reduction of the mental workload under which the ATCOs were operating. On the contrary, as desired, the AA was not activated when workload level was under the threshold, to prevent too low demanding conditions that could bring the operator's workload level toward potentially dangerous conditions of underload.

## Introduction

The main goal of *Human Factor* (HF) studies is to ensure good interactions between the work environment and human capabilities (Wickens, 1992). Humans can adapt themselves to a variety of work environments, performing various tasks, also simultaneously, by using different equipment. Obviously, the greater the number and variety of tasks to perform and devices to use is, the higher the workload experienced is. It has been widely demonstrated that too high operator's mental workload level (*overload*) could result in a degradation of performance and/or an increase in the errors commission probability (Reason, 2000). Therefore, during the past decades, it has been deeply investigated the possibility of developing intelligent systems able to *automatically* support the operator in executing its working tasks, in order to reduce the experienced workload, and consequently keep the performance within high levels, and limit the probability of errors commission.

In general, the term “*Automation*” refers to the process of entirely or partially allocating activities constituting a task usually performed by a human, to a machine, or a system (Parsons, 1985). In particular, Sheridan (1992) identified 10 different *Level of Automation* (LOA), differing in the number and type of activities of the whole task allocated to the operator and to the system, from the Level 1 (operator's fully manual control) to the Level 10 (fully automated control). The first developed form of automation was the *Static Automation* (St-A), i.e., an *on/off* technology active at a fixed LOA, or not active at all. Certainly, the introduction of St-A in operational environments has brought clear benefits (Scerbo, 1996), however research on human interactions with automation has shown that St-A could also introduce several disadvantages, such as monitoring inefficiency, loss of situational awareness, impaired decision making, complacency, and manual skill degradation (Parasuraman et al., 1993). In contrast, systems in which automated aids are implemented dynamically, in response to changing task demands on the operator, may be less vulnerable to such problems. This kind of automation is called *Adaptive Automation* (AA) (Rouse, 1976), recently described as one of the “most important ideas in the history of HF and ergonomics” (Hancock et al., 2013). In particular, an AA-based system is able to adjust continuously the proper LOA, i.e., to assign the authority on specific functions to either the humans or the automated system, depending on the task difficulty and the operator's workload. It has been demonstrated how *Adaptive Automation* is superior to *Static Automation*, since the former is able to ensure operator's workload within the *optimum* range, preserve his/her skill level, guarantee continuous task involvement, and vigilance, thus increasing his/her performance (Rouse, 1988; Wickens, 1992; Byrne and Parasuraman, 1996). Several strategies concerning the triggering mechanism for shifting among modes or levels of automation have been proposed (Scerbo et al., 2003). Three are the main approaches described in the literature: (i) the *Critical-event strategy*, based on the *a-priori* assumption that human workload may become too high when the critical events occur (Hilburn et al., 1997); (ii) the *Performance-measurement strategy*, based on the use of operator's performance during the task itself or additional ones (also called *behavioral measures*) to estimate current and predicted operator's state and to infer whether workload is excessive or not; (iii) *Neurophysiological measurement strategy*, based on the recording of operator's neurophysiological signals, e.g., *electroencephalogram* (EEG), *electrocardiogram* (ECG), *Galvanic Skin Response* (GSR), to infer his actual mental workload (Scerbo et al., 2001). Collectively, each strategy has pros and cons. Although the use of the first two approaches has been successfully employed in several studies, the *neurophysiological measurements-based approach* has several advantages (Byrne and Parasuraman, 1996). Firstly, unlike the *critical-events approaches*, neurophysiological measures could be obtained continuously and online. Secondly, compared with *performance measures*, the neurophysiological ones may be recorded continuously without using overt responses (i.e., additional tasks) and may provide a direct measure of the mental (covert) activities of the operator. Also, neurophysiological measures have higher resolution than performance measures (Di Flumeri et al., 2015). Finally, neurophysiological measures can be used not only to trigger the AA, but also to highlight why AAs are important for enhance the safety in high-risk and high-demanding tasks. This potentially offers new perspectives for adaptive intervention to optimize performance by acting on specific aspects of the operator's behavior.

Byrne and Parasuraman (1996) assessed that the advantage of applying neurophysiological measures in triggering AA was very clear, but the “effective application of psychophysiology in the regulatory role may require years of effort and considerable maturation in technology.” Nowadays, 20 years later, such “effective application” could become reality thanks to the progresses in *Brain-Computer Interfaces* (BCI) research. Briefly, a BCI is defined as “*a system that measures Central Nervous System (CNS) activity and converts it into artificial output that replaces, restores, enhances or improves natural CNS output and thereby changes the ongoing interactions between the CNS and its external or internal environment”* (Wolpaw and Wolpaw, 2012). Such definition summarizes the progresses of the scientific community in this field during the last decades, since at the moment the possibility of using the BCI systems outside the laboratories (Aloise et al., 2010; Blankertz et al., 2010; Aricò et al., 2011; Riccio et al., 2015; Schettini et al., 2015), by developing applications in everyday life is not just a theory but something very close to real applications (Zander et al., 2009; Blankertz et al., 2010; Aricò et al., 2016). This technology has been defined *passive Brain-Computer Interface* (pBCI). In particular, in pBCI technologies, the system recognizes the spontaneous brain activity of the user related to the considered mental state (e.g., emotional state, workload, attention levels), and uses such information to improve and modulate the interaction between the operator and the system itself. Thus, in the context of AA, the pBCIs perfectly match the needs of the system in terms of *Human-Machine Interaction* (Parasuraman et al., 1992; Zander and Jatzev, 2012).

In this context, the most studied mental state is the *Mental Workload* (MWL), due to its strong relationship with the user's performance variations. MWL is a complex construct, generally defined as the actual task cognitive demand related to the real cognitive capacity of the operator (O'Donnell and Eggemeier, 1986). Several empirical investigations have suggested that performance declines at either far ends of the workload demand profile, i.e., when event rates are excessively high (*overload*) or extremely low (*underload*) (Yerkes and Dodson, 1908; Calabrese, 2008). Therefore, it is crucial to have a reliable estimation of the actual mental workload experienced by the operator along the execution of the task, in order to make the user interface able to preserve a proper level of the user's mental workload, avoiding under- or overload state (Hancock and Warm, 1989; Borghini et al., 2012, 2015b). In this regard, neurophysiological techniques have been demonstrated to be able to assess mental workload of humans with a high reliability, even in operational environments (Mühl et al., 2014; Borghini et al., 2015a; Di Flumeri et al., 2015). Many neurophysiological measures have been used for the mental workload assessment, including *Electroencephalography* (EEG), *functional Near-InfraRed* (fNIR) imaging, *functional Magnetic Resonance Imaging* (fMRI), and other biosignals, such as *Electrocardiography* (ECG) and *Galvanic Skin Response* (GSR) (Wood and Grafman, 2003; Ramnani and Owen, 2004; Borghini et al., 2014). Among all these techniques, Aricò et al. (2016) have highlighted the clear advantages of using EEG signal to implement pBCI applications. Several studies, in particular in the aviation domain, have developed efficient EEG-based mental workload indexes. The preliminary results of Brookings et al. (1996) showed that the effects of the task demand were evident on the EEG rhythms variations. EEG power spectra increased in the theta band, while significantly decreased in the alpha band as the task difficulty increased, over parietal and frontal brain sites. More recently, Shou et al. (2012) found that “the frontal theta EEG activity was a sensitive and reliable metric to assess workload […] during an ATC task at the resolution of minute (s).” The same findings have been highlighted by Borghini et al. (2013) involving pilots in flight simulation tasks. In other recent studies involving ATCOs (Aricò et al., 2013, 2014, 2015b,c; Borghini et al., 2014; Di Flumeri et al., 2015; Toppi et al., 2016), it was demonstrated how it was possible to compute an EEG-based Workload Index able to significantly discriminate the workload demands of the ATM task, and to monitor them continuously by using frontal-parietal brain features. Other studies about the mental workload estimation by using neurophysiological indexes, have been proposed also in other operational contexts (Car drivers - Kohlmorgen et al., 2007; Borghini et al., 2012a; military domain - Dorneich et al., 2005).

Despite the scientific evidences on the possibility of measure the mental workload by using neurophysiological measures, and of using them to trigger AA solutions (i.e., pBCI), only few examples have been proposed in this regard, the most of them in laboratory settings. The concept of a “closed-loop system,” i.e., the mitigation of an operator's level of workload through a closed-loop system driven by the operator's own EEG, was theorized during the past decade (Prinzel et al., 2000; Schmorrow et al., 2006). Freeman et al. (1999) proposed one of the first EEG-based studies about the impact and efficiency of AA: they developed an application able to switch between automatic and manual mode of the tracking task of the *Multiple-Attribute Task Battery* (MATB, Comstock, 1994) by adopting EEG indexes based on the Theta and Alpha band power spectra over the parietal cortex, according to the results obtained in a precedent study (Pope et al., 1995). A similar study was also proposed by Prinzel et al. (2000). Both the studies highlighted a significant decrement in the mental workload experienced by the users by activating the automation solutions, confirmed by using both the EEG-based indexes and the subjective measures. Also, Berka et al. (2005) studied a similar application by using the Aegis simulator, i.e., a military simulation environment. Also in this study it has been highlighted the possibility of monitoring in real-time the mental workload and to use EEG-based indexes to reallocate tasks and system aids. However, as stated before, such studies were performed in laboratory settings. Recently, Abbass et al. (2014) built an adaptive controller's working position based on cues extracted from EEG signals and task complexity indicators from the scenario, demonstrating that four operators achieved a better performance while the AA was activated. Apart from such preliminary studies, no evidences about the possibility to use pBCI technologies to realize AA systems in real settings have been proposed.

In this study, we present a passive-BCI system fully integrated with a high realistic ATM simulator able to trigger adaptive solutions in real-time depending on the mental workload estimated by means of the ATCO's brain activity. We expected that the pBCI system would be able to trigger the AA solutions and to reduce the task workload demand in order avoid both under and overload conditions.

## Materials and methods

### Subjects

Twelve *Air Traffic Controller* (ATC) students (23 ± 2 years old) from the É*cole Nationale de l'Aviation Civile* (ENAC, Toulouse, France, one of the most important training schools for ATCOs and Pilots in the World) have been involved in this study. They were selected in order to have a homogeneous experimental group in terms of age and expertise. These students were finishing their 3 years training at ENAC. The experiment was conducted following the principles outlined in the Declaration of Helsinki of 1975, as revised in 2000. It received the favorable opinion from the Ethical Committee of the Sapienza University of Rome, Dept. Physiology and Pharmacology. The study involved only healthy, normal subjects, recruiting on a voluntary basis. Subjects were free to accept or not to take part to the experimental protocol. All the recruited subjects accepted to participate to the study. Informed consent was obtained from each subject on paper, after the explanation of the study. No other individual information apart from the cerebral activity was gathered for the purpose of this study. Only aggregate information has been released while no individual information were or will be diffused in any form.

### Experimental protocol

The subjects have been asked to manage a functional interface that simulates a high realistic ATM scenario. The complexity of the task could be modulated according to how many aircrafts the ATCO had to control, the number and type of clearances required over the time and the number/trajectory of other interfering flights. Also, for the purposes of this study, specific AA solutions have been embedded in the ATM interface, with the aim to induce a decreasing in the operators' mental workload during high workload situations. These AA solutions were the result of several brainstorming sessions with subject matters experts (senior ATCOs), human factor and human computer interaction specialists. According to design principles described in the Introduction Section, few realistic proposals have been made and implemented. Those AA solutions have been described in Table 1.

**Table 1:** **Description of the AA solutions developed at ENAC**.

**AA solution**	**Description**
Adapt Situation Awareness Monitoring by reducing or removing alerts	The monitoring agent sends all alerts to the controllers not considering the controller's workload, traffic complexity or the alert emergency. The interface could filter those alerts to prevent distracting the controller with an alert which is not critical if the controller's workload is high.
	High workload: Only critical alarms are shown to the controller.
	Low workload: No alarms
Highlighting of calling station	Aircraft labels on the radar image are highlighted to help controllers locate the aircraft currently speaking on the radio.
	High workload: the background of the classing of a calling station is blue and it remains as it is until the controller moves the mouser pointer over the aircraft.
	Low workload: no highlight
Adapt Short Term Collision Avoidance (STCA) alert design	The graphical design of the STCA is not the most efficient to catch controller's attention. The design could be changed to alert the controller faster. An animated box around the label will reduce the perception time of the controller.
	High workload: graphical design used is box animation (a box appears around the label with some margin and shrinks until no margin is left)
	Low workload: graphical design used is color blinking
Reduce visual load	Reduce visual load by removing non relevant aircraft for the sector.
	High workload: only aircraft that will cross or are in the controlled sector are displayed on the screen.
	Low workload: all aircraft are displayed.

Such ATM interface has been developed and hosted at ENAC.

**Figure 1:**
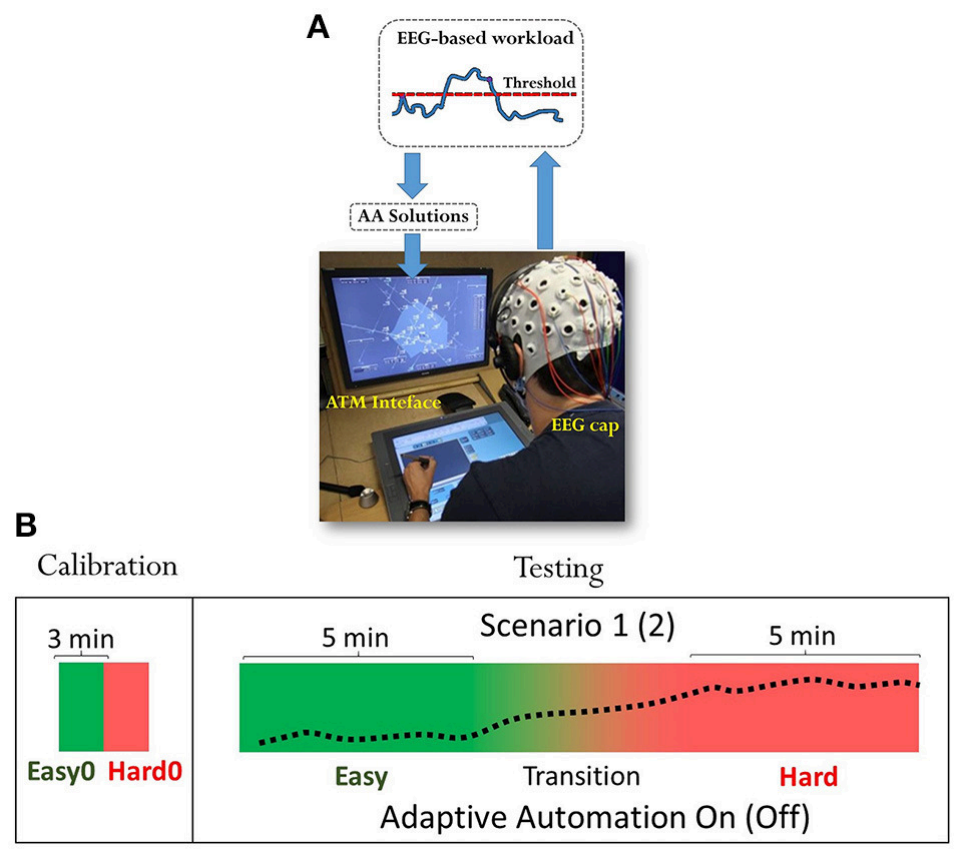
**(A)** figure shows ATCO students wearing the EEG cap during the experiment and managing the ENAC platform, composed of two screens, a 30″ (RADAR) screen to display radar image and a 21″ screen to interact with the radar image (ATM interface). The mental workload of the user was evaluated online and specific AA solutions changed online the behavior of the RADAR screen depending on the actual mental workload level. **(B)** ATCO students have been asked to perform two ATM scenarios, one in which adaptive solutions could be triggered by the EEG mental workload index (AA On), and the other one in which adaptive automation has been disabled (AA Off). Presentation of each scenario and condition has been randomized to avoid any habituation and expectation effects.

Electroencephalogram (EEG) signal has been recorded and used online to evaluate the mental workload of the controllers. Such mental state has been used to trigger the RADAR screen interface by using the AA solutions described previously, only when the workload of the user become higher than the threshold defined during a specific calibration phase. Triggers depending on the actual mental workload of the user has been sent to the ATC interface by using a dedicated middleware developed at ENAC. Figure 1A shows the platform architecture realized for the purpose of such experiment.

ATCO students that took part to the experiment were already well trained to use such interface. In particular, controllers have been asked to handle with two ATM scenarios under two different conditions (Easy and Hard). One scenario in which the AA could be triggered by the EEG-based mental workload index of the user (*AA On*), and the other one in which the operators' EEG-based mental workload index has been computed and stored, but not used to trigger the ATM interface (*AA Off*). Each scenario lasted 15 (min), the first 5 and the last 5 (min) have been designed to keep the task difficulty as constant as possible, low (Easy), and high (Hard), respectively. The middle part of the task (5 min) has been designed to simulate a realistic transition between the Easy and Hard segments, but it has not been used in the analysis (Figure 1B). The two scenarios have been designed comparable in terms of complexity within the same difficulty levels (e.g., *Easy* of Scenario 1 and *Easy* of Scenario 2). The combinations of scenarios and conditions (AA On/Off) have been randomized to avoid any habituation effect and bias in the results. In addition, ATCO students have been asked to perform two traffic samples of 3 (min) before the execution of the experimental scenarios, respectively, easy (*Easy 0*), and hard (*Hard 0*), to be used for the calibration of the EEG-based workload algorithm. Let us name such tasks of 3 (min) as “Calibration scenarios” and the two consecutive 15 min-long scenarios as “Testing scenarios.” At the end of each Testing scenario, Controllers have been asked to fill the NASA-TLX questionnaire (Hart and Staveland, 1988), in order to evaluate their perceived mental workload in performing the different conditions (AA On/Off). NASA-TLX is a widely-used, multidimensional assessment tool that rates subjective perceived workload between 0 and 100.

#### EEG-based online mental workload classifier

##### Signals recording

For each subject, scalp EEG signal was recorded (g.USBamp, gTec, Austria) from 9 Ag/AgCl wet electrodes (Fpz, Fz, F3, F4, AF3, AF4, Pz, P3, P4) at 256 (Hz), referenced to both the mastoids and grounded to the Cz electrode, according to the 10–20 International System (Jurcak et al., 2007).

##### Processing

The recorded EEG signal has been band-pass filtered (1÷30 (Hz), 5th order Butterworth filter) and the Fpz channel has been used to remove eyes-blink artifacts from the EEG data by using the regression-based algorithm REBLINCA (Di Flumeri et al., in press). With respect to other regressive algorithms (e.g., Gratton method, Gratton et al., 1983) the REBLINCA algorithm has the advantages to preserve EEG information in blink-free signal segments by using a specific threshold criterion that recognize automatically the occurrence of an eye-blink, and only in this case the method correct the EEG signals. If there is not any blink, the method has not any effect on the EEG signal. In addition, the REBLINCA method does not require EOG signal(s). The band-pass filtered (1÷7 (Hz), 5th order Butterworth filter) Fpz signal has been used as template to remove eye-blinks contribution from the EEG signal. Regressive weights for each EEG channel were calculated on the Calibration scenarios and have been used both offline (in the same scenarios) and even online in the Testing scenarios. This step has been performed because the eye-blinks contribution could affect the frequency bands related to the mental workload, in particular the theta EEG band. At this point, the EEG signal has been segmented into epochs of 2 (s), shifted of 0.125 (s). For other sources of artifacts (i.e., ATCOs normally communicate verbally and perform several movements during their operational activity), specific procedures (Threshold criterion, Trend estimation, Sample-to-sample difference) available in the EEGLAB toolbox (Delorme and Makeig, 2004) have been applied. In particular, in the Threshold criterion the EEG epochs are marked as “artifact” if the EEG amplitude is higher than ±100 (μV). In the trend estimation, the EEG epoch is interpolated in order to check the slope of the trend within the considered epoch. If such slope is higher than 3 (μV/epoch), the considered epoch will be marked as “artefact.” The last step calculates the difference between consecutive EEG samples. If such difference, in terms of amplitude, is higher than 25 (μV), it means that an abrupt variation (no-physiological) happened, thus it will be marked as “artefact.” Hence, the epochs marked as “artifact” were totally rejected.

##### Algorithm calibration

As stated before, the Calibration scenarios (*Easy 0* and *Hard 0*) have been used to calibrate the algorithm before the Testing scenarios presentation. In particular, the *Power Spectral Density* (PSD) of EEG epochs related to each calibration scenario (*Easy 0* and *Hard 0*) has been calculated by using only the frequency bands directly correlated to the mental workload (frontal theta and parietal alpha bands). The EEG frequency bands [frequency resolution of 0.5 (Hz)] of interest have been defined for each ATCO by the estimation of the *Individual Alpha Frequency* (IAF) value (Klimesch, 1999; Babiloni et al., 2000). At this point, the classification algorithm *automatic stop Stepwise Linear Discriminant Analysis* (asSWLDA, patent number P1108IT00, Aricò et al., 2015a, 2016) has been used to identify the most relevant discriminant features among the different experimental conditions (i.e., *Easy 0* and *Hard 0*), related to the lowest and the highest task complexity. Once identified, the asSWLDA classifier assigns to each significant feature specific weights (*w*_*i train*_), plus a bias (*b*_*train*_). On the contrary, weights related to those features not relevant for the classification model are set to “0.” These parameters have been used later on to compute online the mental workload index of the user during the Testing scenarios.

##### Stepwise linear discriminant analysis (SWLDA)

The SWLDA regression consists in the combination of the forward and the backward stepwise analyses, where the input features are weighted by using ordinary least-squares regression to predict the target class labels. The method starts by creating an initial model of the discriminant function in which the most statistically significant feature is added to the model for predicting the target labels (pval_ij_ < α_ENTER_), where pval_ij_ represents the *p*-value of the ith feature at the jth iteration (in this case the first iteration). Then, at each new iteration, a new term is added to the model (if pval_ij_ < α_ENTER_). If there are not more features that satisfy this condition, a backward elimination analysis is performed to remove the least statistically significant feature (if pval_ij_ > α_REMOVE_) from the model. This process goes on unless there are no more features satisfying the entry (α_ENTER_) and the removal (α_REMOVE_) conditions (Draper, 1998), or until a predefined number of iterations is reached (Iteration_MAX_). Normally, it is possible to optimize a SWLDA regression by tuning all or some of the three parameters available in the algorithm (α_ENTER_, α_REMOVE_, and Iteraction_MAX_). There are not standard procedures to choose these parameters, and in theory, they should be easily manually (empirically) gauged based on the expected characteristics of the data.

The standard SWLDA algorithm uses α_ENTER_ = 0.05 and α_REMOVE_ = 0.1, and no constrains on the Iteraction_MAX_ parameter are imposed. For summarize, the standard training process goes on unless there are no more features satisfying the entry (α_ENTER_) and the removal (α_REMOVE_) conditions.

##### Automatic stop stepwise linear discriminant analysis (asSWLDA)

The SWLDA is one of the best outperforming linear classifiers (Craven et al., 2006; Aloise et al., 2012), in fact with respect to other linear methods it has the advantage of having automatic features extraction, so that insignificant terms are statistically removed from the model. Despite the strength of the method, it is simple to realize how it would be difficult to set up properly the right parameters in order to optimize the algorithm. In fact, it is expected that the more general the classification training would be, the higher the reliability of the algorithm over time will be (Vapnik, 2000). For example, if the chosen parameters are too selective (α_ENTER_ and/or α_REMOVE_, and/or Iteration_*MAX*_ values too much low), maybe the features added to the model will be not sufficient for predicting the target labels (underfitting, von Luxburg and Schoelkopf, 2011). On the contrary (α_ENTER_ and/or α_REMOVE_, and/or Iteration_MAX_ values too high), most of the features added in the final model could be related to spurious differences between classes of the training set, that are obviously not generalizable, so that, the reliability of the algorithm decreases over time (overfitting, Vapnik, 2000). The reduction of features selected by the classifier in general could mitigate the overfitting.

The optimum solution to these problems would be to find out a criterion able to automatically stop the algorithm iterations when the best number of features (#Features_*OPTIMUM*_) have been added to the model. In the following, it will be reported a modified version of the standard SWLDA algorithm that encloses this “automatic stop” criterion described previously. The name of this implementation is automatic-stop Stepwise Linear Discriminant Analysis (asSWLDA, Aricò et al., 2015a, 2016).

**Figure 2:**
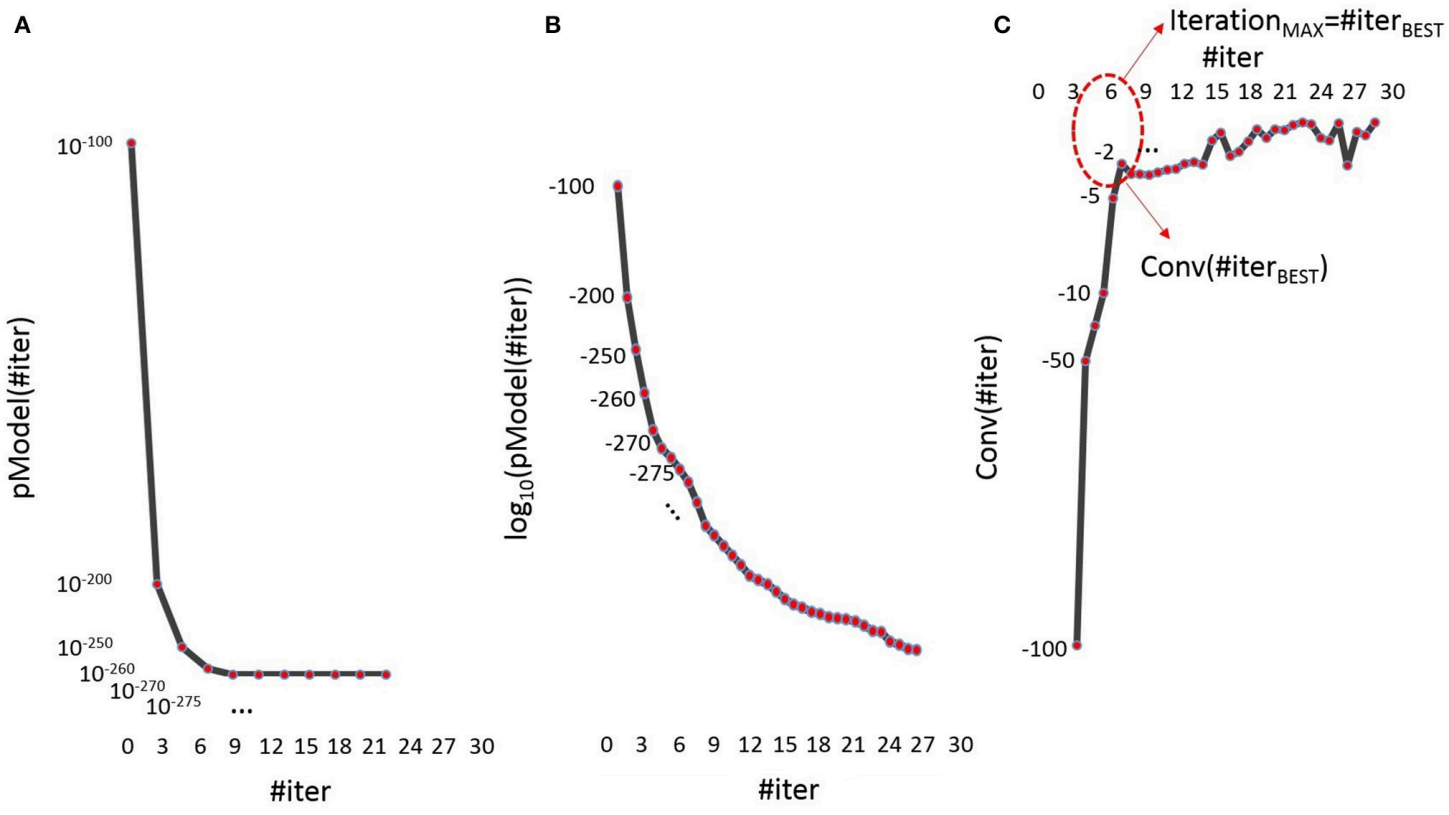
**Representation of the (A) pModel vector, the (B) log10 of the pModel vector and the (C) Conv function for each iteration, for a representative subject**. In particular, in the figure **(C)** there are also showed (i) the Conv(#iterBEST), in other words the lower distance of the Conv(#iter) function from the point (0,0) and (ii) the correspondent IterationMAX, that is #iterBEST.

As we stated above, the tuning parameters in the standard SWLDA algorithm are three: α_ENTER_, α_REMOVE_, Iteration_MAX_. In this implementation, the first two parameters are left unbind as in the standard SWLDA implementation (i.e., α_ENTER_ = 0.05, α_REMOVE_ = 0.1). In fact, because of the probability (pval_ij_) associated to each feature is strictly related to the actual iteration (in other words, to all the actual features in the models) this probability changes iteration by iteration, and it would result very difficult to impose a condition by using α_ENTER_ and α_REMOVE_. In addition, even if no constrains on the α_ENTER_ and the α_REMOVE_ parameters are imposed, the features would be included in the model in order of significance (i.e., the first feature in the model will be the most significant one, and so on). On the contrary, the value of the Iteration_MAX_ parameter will affect the reliability of the classifier over time (optimum classifier, underfitting, or overfitting). As we stated before, this parameter should be chosen such that #Features_UNDERFITTING_ < < #Features_OPTIMUM_ < < #Features_OVERFITTING_. In order to make the classifier able to find automatically the best IterationMAX parameter, we took into account the *p*-value of the model (pModel), parameter available in the output of the standard SWLDA implementation, that gives information about the global significance of the model at the iteration jth. The more the number of iterations increases (the more features are added to the model), the more the pModel value decreases (tending to zero) with a decreasing exponential shape. First of all, we collected the pModel values for all the iterations [pModel(#iter), Figure 2A]. At this point, we calculated the log10 of the pModel vector [log_10_[pModel(#iter)], Figure 2B], and then the first-order differences between adjacent pModel elements (Equation 1) that we called Convergence function, or Conv(#iter). We used the log10 function since we would have information about the size of pModel order, and after that about the differences between these pModel orders.

Finally, we plotted this vector as a function of #iter (Figure 2C).

(1) $$\begin{array}{l} Conv\left(\#iter\right)\;=\;\log_{10}\left(pModel\left(\#iter+1\right)\right)\\ -\log_{10}\left(pModel\left(\#iter\right)\right) \end{array}$$

We identified as the best Iteration_*MAX*_'s value the number of iterations at which the Conv(#iter) assumed the lowest distance from the point (0,0), plus one (because we are working on the first-order differences, Formulas 2, 3).

(2) $$\begin{array}{rcl} ||Conv\left(\#iter_{BEST}\right)||\; & = & \;\min||Conv\left(\#iter\right)|| \end{array}$$

(3) $$\begin{array}{rcl} Iteration_{MAX}\; & = & \;\#iter_{BEST}+1 \end{array}$$

In fact, the best condition would be to have the least possible features and at the same time the convergence of the model (Formula 4).

(4) $$\begin{array}{l} \log_{10}\left(pModel\left(\#iter+1\right)\right)-\log_{10}\left(pModel\left(\#iter\right)\right)=0 \end{array}$$

*Online EEG-based workload index (W*_*EEG*_*) assessment:* The procedures described above have been applied ongoing with the execution of the testing scenario. To summarize, the band-pass filtered EEG signal has been buffered online in 2 (s) epochs shifted of 0.125 (s). EEG epochs have been cleaned off by eye-blinks contributions and those affected by other sources of artifacts have been discarded. At this point, PSD of the remaining EEG epochs have been calculated by using frontal theta and parietal alpha bands.

Thereafter, the classifier parameters estimated during the calibration phase (w_*i train*_ and b_*train*_) have been used to calculate online the *Linear Discriminant Function* [*y*_*test*_*(t)*, Equation 5], defined as the linear combination of the testing spectral features [PSD calculated by using frontal θ and parietal α bands, *f test* (*t*)] and the classifier weights (*w*_*i train*_), plus the bias (*b*_*train*_).

Finally, a moving average of *n* seconds (nMA) has been applied to the y_test_(t) function in order to smooth it out by reducing the variance of the measures, and the result has been named *EEG-based workload Index* (W_*EEG*_, Equation 6). The higher is the *n* value, the less the variance of the measure will be. For a proper evaluation of the mental workload during the execution of ATM tasks, the *n* value has been set to 30 (s). This value has been chosen together with ATCO and human factor experts, in order to find out the best trade-off between providing a proper workload resolution and, at the same time, an adequate commutation rate of the AA. In addition, in literature it has been shown that EEG is a viable source of information regarding the workload of a person, enabling 95% accuracy when using about 30 s of signal (Gevins et al., 1998).

(5) $$\begin{array}{rcl} y_{test}\left(t\right) & = & \sum_{i}w_{i_{train}}*f_{i_{test}}\left(t\right)+b_{train} \end{array}$$

(6) $$\begin{array}{rcl} W_{EEG}\; & = & \;nMA\left(y_{test}\left(t\right)\right) \end{array}$$

i = # of spectral features; t = [1,2,…,# of EEG epochs]; n = 30 (s).

*Online W*_*EEG*_ *classification:* The *mental workload index* (W_*EEG*_) has been classified online in two classes (HIGH and LOW) to trigger the AA. In particular, if the W_*EEG*_ was higher than a specific threshold, the mental workload of the user was classified as HIGH and the adaptive solutions would be activated. On the contrary, the adaptive solutions were disabled (LOW class). In this regard, a proper connection has been created with the ATM interface, by using the TCP/IP network standard protocol in order to exchange messages (e.g., time frame of the experiment, workload index, classification result, etc.) in real-time. The classification threshold (Class-Threshold) was obtained through a procedure that relies on the use of *Receiver Operating Characteristic* (ROC) curves (Bamber, 1975). This methodology allows to estimate the performance of a binary classifier as its threshold is varied, by representing the *true positive rate* (TPR) against the *false positive rate* (FPR, Bamber, 1975). In this regard, for each subject a *k-fold cross-validation* (*k* = 10, McLachlan et al., 2005) has been performed on the spectral features dataset related to the Calibration scenarios [PSD calculated by using frontal theta and parietal alpha bands, *f*^*j*^(*t*)]. In particular, all the possible (k-1) subsamples have been used to train the asSWLDA classifier, and the related parameters ($w_{i}^{j},\;b^{j}$) have been applied on the remaining subsample to compute the *Linear Discriminant Function* [ *y*^*j*^(*t*), Formula 7]. All the *y*^*j*^(*t*) values related to the *k* iterations, have been stacked in the *Y* vector (Formula 8).

(7) $$\begin{array}{rcl} y^{j}\left(t\right)\; & = & \;\sum_{i}w_{i}^{j}\;*\;f_{i}^{j}\left(t\right)+b^{j} \end{array}$$

(8) $$\begin{array}{rcl} Y & = & {⋃}_{j}{⋃}_{t}y^{j}\left(t\right) \end{array}$$

i = # of spectral features; j = [1,2,…,k]; t = [1,2,…,# of EEG epochs].

This vector (*Y*) and the related labels vector (containing information if the specific element of the *Y* vector is related to the *Easy0* or *Hard0* calibration scenario) have been used to compute the ROC curve. To select the best threshold (Class-Threshold) we used the “minimum distance” method, explained in details in Fawcett (2006). In other words, we selected as Class-Threshold for each subject the *Y* value such that the FPR had been as low as possible, and at the same time the TPR as high as possible.

**Figure 3:**
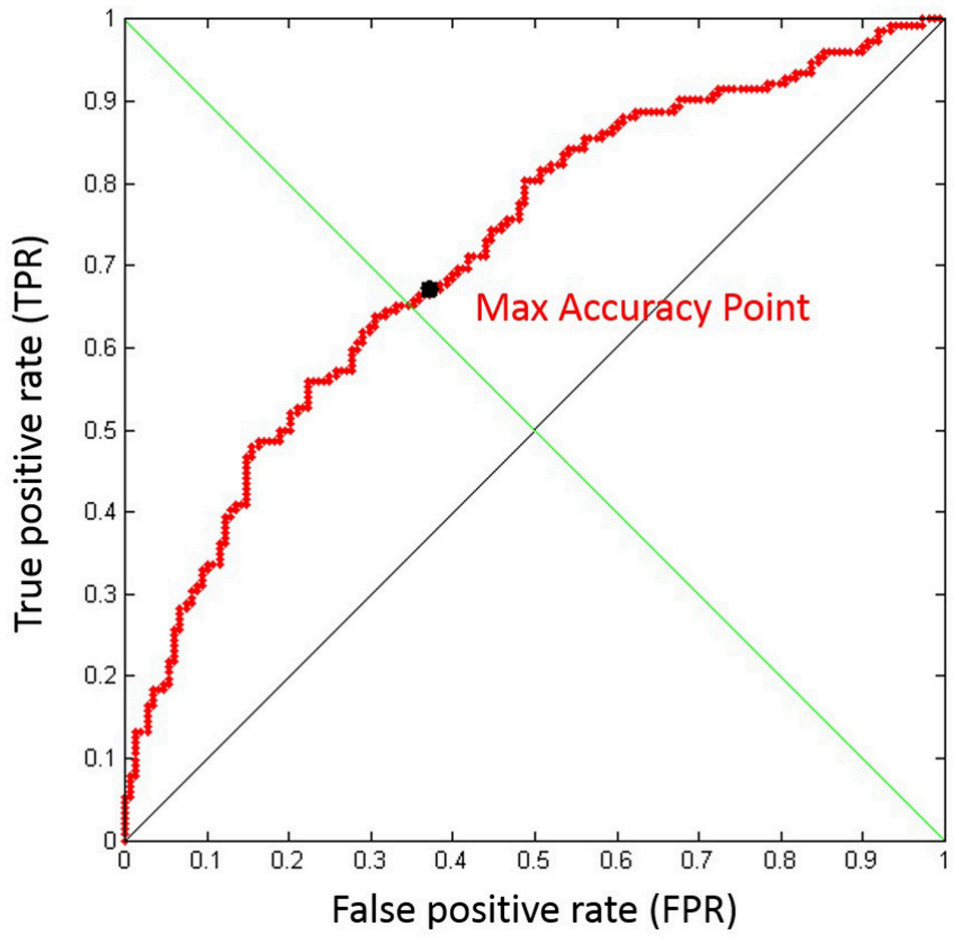
**Graphical representation of the averaged Receiver Operating Characteristic (ROC) over all the subjects related to the offline k-fold cross-validations performed to find the best threshold**. The achieved offline classification performance was of 75 ± 10%. The mean threshold value was 0.48 ± 0.07.

In the following, it has been reported the graphical representation of the averaged ROC over all the subjects related to the offline *k-fold* cross-validations performed to find the best threshold. The mean offline classification accuracy achieved was 75 ± 10%. The mean threshold value was 0.48 ± 0.07 (Figure 3).

### Performed data analyses

The following analyses had the aim to demonstrate the two hypothesis of the present study:

1. it is possible to trigger the AA by using the online recognition of the actual mental workload of the user,
2. the AA induces a reduction of the mental workload of the operator when it became high and consequently an increasing in performances execution of the task.

##### Triggering of adaptive solutions

To demonstrate the first hypothesis, we compared the number of times in which the W_EEG_ activated the adaptive solutions in the Easy and Hard slots during the *AA On* condition, by using a two-tailed paired *t*-test (α = 0.05). In fact, we expected that the mental workload classifier was able to induce an activation of the AA more often during the Hard period with respect to the Easy one.

To investigate the second hypothesis, we performed analyses on subjective and neurophysiological workload measurements along the two conditions (AA On/Off) and difficulty levels (Easy and Hard), and behavioral performances achieved by the operators in the two conditions.

##### Subjective workload assessment

A two-tailed paired *t*-test (α = 0.05) has been performed on the NASA-TLX scores to investigate differences between the two AA conditions (AA On/Off) in terms of workload perception.

##### Behavioural performance assessment

The ATM interface recorded information about the reaction time and the number of airplanes the ATCO had assumed in each specific task condition (AA On/Off). Together with ATCO and human factor experts we defined an index based on these parameters related to the performance achieved by the operator during the execution of the task. In the following all the considered parameters used to assess the performance of the operator have been reported:

- Time Shoot: the interaction time needed for delivering an aircraft to the next sector,
- Time Route: the interaction time to display the graphic route of an aircraft,
- Time Cancel: the interaction time between triggering write recognition box and pressing of the cancel button,
- Time Annul: the interaction time between the opening of a pie menu or write recognition box and clicking on the radar image background,
- Time Turn: the interaction time between triggering the menu and the validation of the write recognition box,
- Time Flight Level: the interaction time between triggering the menu and the validation of the write recognition box,
- Time Direct: the interaction time between triggering the menu and selecting the waypoint in the flight plan list.

Since the ATCOs might adopt different strategies to manage the air-traffic, their reaction times have been normalized on the number of airplanes assumed in the different phases of the simulation. The performance index, *Weighted Mean Reaction Time* (WMRT), has then been defined as the average of the weighted reaction times described previously.

A two-tailed paired *t*-test (α = 0.05) has been performed between WMRT indexes over the two conditions (AA On/Off).

##### Neurophysiological workload assessment

We compared the W_*EEG*_ indexes referred to the two difficulty levels (Easy, Hard) *within* and *between* the two AA conditions (AA On/Off). In particular, we performed 4 two-tailed paired *t*-test (α = 0.05) to compare difficulty levels *within* the same AA condition (i.e., AA On: Easy vs. Hard; AA Off: Easy vs. Hard), and to compare the two AA conditions *within* the same difficulty level (Easy: AA On vs. AA Off; Hard: AA On vs. AA Off).

Before every statistical analysis, the *z-score* transformation (Zhang et al., 1999) has been used to normalize the data.

## Results

### Subjective workload assessment analysis

**Figure 4:**
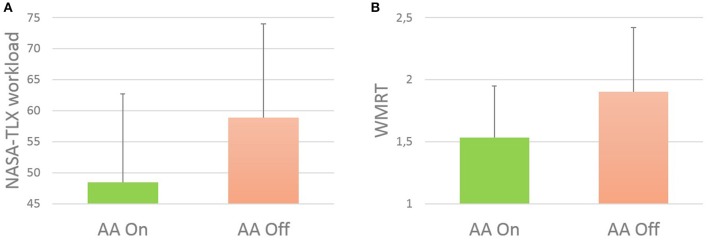
**(A)** Vertical bars related to the subjective measure of the mental workload of the ATCOs, by using the NASA-TLX questionnaire. The results showed a not significant trend (*p* = 0.068) between the two conditions (AA On and AA Off). **(B)** Vertical bars related to the Weighted Reaction Time index (WMRT), reflecting behavioral performances of operators during the two conditions (AA On and AA Off). The results showed a significant (*p* = 0.045) increasing of task performances execution during AA On condition.

The *t*-test results showed that the perceived workload of the user during the *AA Off* condition was not significantly higher (*p* = 0.068) in comparison to the *AA On* condition. It has to be underlined that the workload scores provided by the subjects referred to the whole AA condition, composed by the Easy, the Hard and the transition portion (Figure 4A).

### Behavioural performance assessment

The *t*-test results showed that performances of operators during the AA On condition were significantly (*p* = 0.045) higher (WMRT index lower) than performances in the AA Off condition (Figure 4B). Of course, higher reaction times reflect lower performances, and vice versa.

### Neurophysiological workload analysis

**Figure 5:**
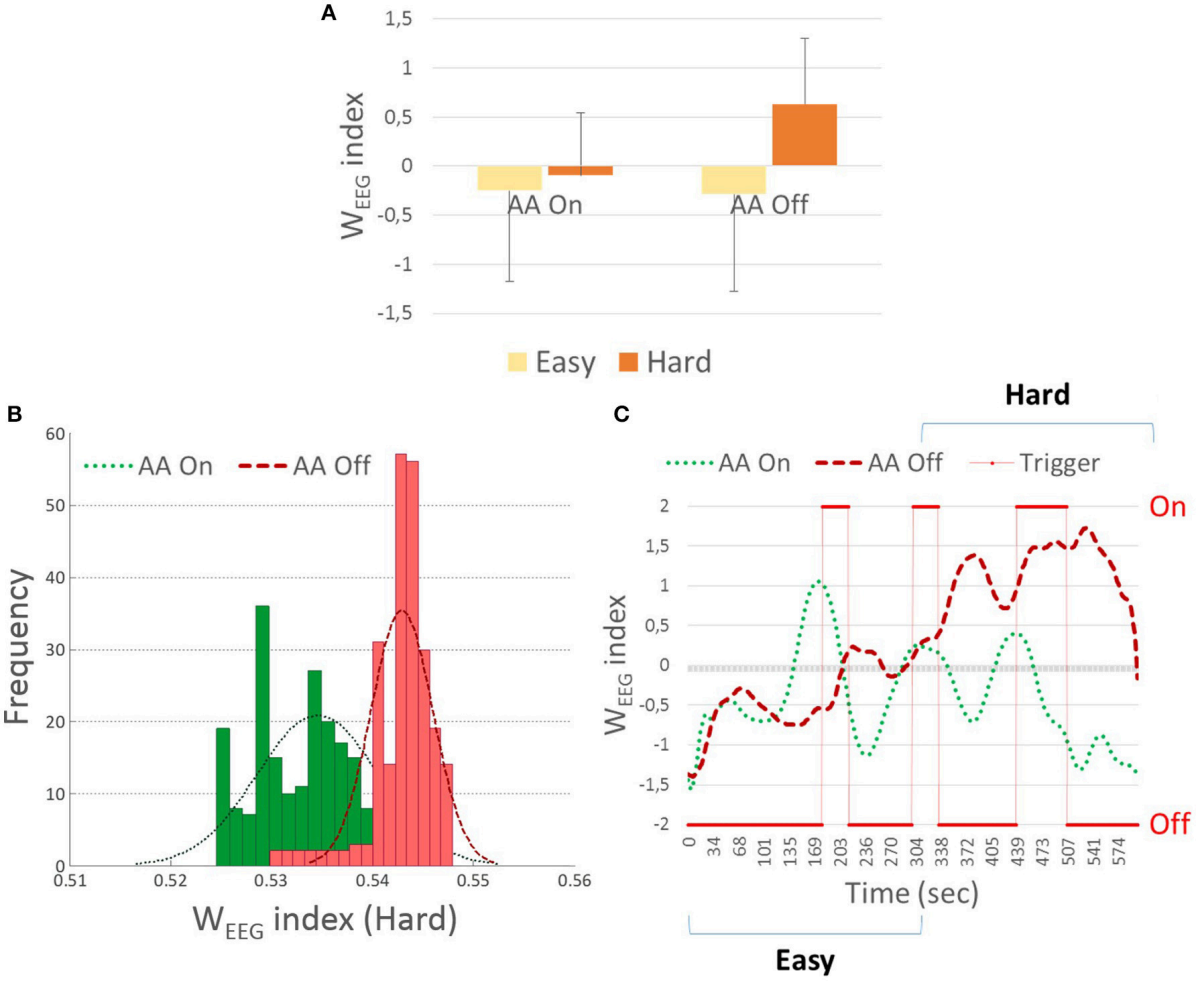
**(A)** Vertical bars of the neurophysiological workload index distributions (W_*EEG*_) related to the *Easy* and the *Hard* slots, during the conditions *AA On* and *AA Off*. **(B)** Figure shows the shape of the W_*EEG*_ distributions related to the Hard slot, for both the two conditions (*AA On/Off*). **(C)** Figure shows the time course of the W_*EEG*_ index related to the Easy and Hard slots, in both the two conditions (*AA On/Off*) together with the AA activation segments (Trigger) for a representative subject. The figure suggests that when the AA is activated, the W_*EEG*_ index related to the *AA On* condition decreases accordingly.

The *t*-tests showed a significant increasing (*p* = 0.03) of the W_*EEG*_ indexes distribution between the *Easy* and the *Hard* periods only for the *AA Off* condition. On the contrary no significant differences (*p* = 0.65) have been highlighted between the W_*EEG*_ indexes related to the *Easy* and the *Hard* slots during the *AA On* condition (Figure 5A). In addition, a significant increment (*p* = 0.04) of the W_*EEG*_ indexes distributions related to the *Hard* slot of the *AA Off* condition with respect to the *Hard* slot of the *AA On* condition has been reported. In this regard, Figure 5B shows the shape of the W_*EEG*_ distributions related to the *Hard* slot, for both the two conditions (*AA On/Off*). Instead, no significant trends (*p* = 0.95) have been highlighted between the *Easy* slots of the two AA conditions. In conclusion, Figure 5C shows the time course of the W_*EEG*_ index related to the *Easy* and *Hard* slots, in both the two conditions (*AA On/Off*) together with the AA activation segments (Trigger) for a representative subject. The figure suggests that when the AA is activated, the W_*EEG*_ index related to the *AA On* condition decreases accordingly.

In conclusion, the *t*-test results showed that the number of AA activations triggered by the W_*EEG*_ index (*AA On* condition) was significantly lower (*p* = 0.04) during the Easy period with respect to the Hard one. In other words, the classifier triggered more often the ATM interface when the operator's workload was classified as HIGH (i.e., during the Hard period).

## Discussion

In the present study, we proposed a pBCI system able to evaluate and classify online the operators' mental workload by using the EEG activity (W_*EEG*_). Depending on the classification result (LOW or HIGH), the system was able to trigger online the operator's interface, changing its behavior by activating or deactivating Adaptive Automation (AA) solutions. The system has been integrated in an already existing ATM experimental platform, developed at ENAC. Twelve ATCO students have been asked to test the system by managing high realistic ATM scenarios under different difficulty levels. In particular, we expected that the proposed system was able: (i) to trigger in the right way the ATM interface (i.e., the number of times in which the system activates the AA should be higher during the *Hard* scenario period in respect to the *Easy* one in order to prevent overload situation during the former and underload during the latter), and (ii) to induce a decreasing of the mental workload perceived by the operators when the adaptive solutions were activated and consequently an increasing in task performances execution.

Regarding the first point, results confirmed that the number of AA activations were significantly (p = 0.04) higher during the Hard scenario period with respect to the easy one. The behavior of activating AA solutions only when the workload of the operator becomes high is an important issue. In fact, if the AA solutions were improperly or even always activated, the workload of the user could decrease too much, and, in general, induce performance reduction and decreasing safety. In this regard, results showed no differences in terms of mental workload between the *Easy* scenarios related to the AA On and AA Off conditions.

For the second point, results revealed a significant decreasing of the mental workload of the operator when the proposed pBCI system activated the AA solutions (*AA On* condition) with respect to the condition without AA solutions (*AA Off*). Furthermore, in the *AA On* condition, the *EEG–based mental workload index* (W_*EEG*_) related to the *Hard* task was not significantly higher than the *Easy* related workload condition. On the contrary, when the AA solutions were not activated (*AA Off* condition), the *Hard* related mental workload was significantly higher than the *Easy* one. This behavior was confirmed also by the subjective measures, in particular, the NASA-TLX questionnaire revealed an increasing of the perceived mental workload when the AA solutions were not activated (*AA Off*) with respect to the scenarios in which the pBCI could activate the AA solutions (*AA On*). Finally, behavioral performance analysis also revealed that AA On condition induced a significant increment in task performance with respect to the AA Off condition.

The results achieved in this study, performed in a high-realistic setting, confirmed the findings of other similar works. For example, Freeman et al. (1999) have proposed an application consisting in the switching between automatic and manual mode of the tracking task of the *Multiple-Attribute Task Battery* (MATB, Comstock, 1994) by adopting EEG indexes based on the Theta and Alpha band power spectra over the parietal cortex. A similar study was also proposed by Prinzel et al. (2000). Another application was performed by Berka et al. (2005) by using a military simulation environment triggered by an EEG–based workload index. All the studies have highlighted a significant decrement in the mental workload experienced by the users by activating the automation solutions. Anyhow, it has to be stressed that such studies have been performed in laboratory settings, where the environment and the difficulty levels used in the proposed tasks were very under control.

On the contrary, in the present study, we confirmed the same findings, but in realistic settings where difficulty levels were not constant but changed over time reflecting real traffic scenarios, and the operators could communicate and move as they normally do during real work shifts. In conclusion, despite the possible presence of artifacts coupled to the EEG signal, and the realistic shape of the traffic samples, the significance of the results is remarkable.

The only study performed in a more realistic environment was proposed by Abbass et al. (2014), in which four ATC experts had to manage a simulator for 50 min, while both EEG and traffic indicators were used in a rule-based system, which decided if there was the need to activate adaptation (AA) or not. In particular, the EEG index was based on the Theta to Beta ratio over the whole brain scalp. The results indicated that the 4 subjects perceived an overall increasing in their performance (assessed by questionnaire) when the AA was enabled, but this result was not statistically significant. In addition, no significant differences have been highlighted between complexity indexes (estimated from task parameters) when the AA was enabled or disabled.

## Conclusion

The aim of the study was to investigate the possibility of using information coming from the operators' brain activity (i.e., mental workload) to realize a p-BCI system able to trigger specific Adaptive Automation solutions in Air Traffic Management contexts. The results demonstrated that the proposed pBCI system was able to (i) differentiate workload levels related to different difficulty tasks (i.e., *Easy* and *Hard*), (ii) trigger the AA solutions mostly when the workload of the operator was high, so preventing overload and underload situations. Also, it has been demonstrated that the whole AA system was able to (iii) induce a significant reduction of the workload level experienced by the operator during the execution of the ATM task and iv) a significant increasing of the task performance execution.

Thanks to the promising results, further experiments will be performed to investigate the possibility to develop AA solutions triggered by using more than two states (i.e., workload HIGH and LOW), in order to have a more specific *Dynamic Function Allocation*. Ideally, it should be possible to modulate all the 10 levels of LOA (Sheridan and Verplank, 1978) depending on the actual mental workload of the user.

## Author contributions

PA wrote the paper and developed part of the online system to measure the mental workload of the user. In addition he took part to the definition of the experimental protocol. GB and GD made offline data analysis and related statistics. In addition they contributed to the writing process and to the protocol definition and data acquisition. AC contributed to the revision of the manuscript. SB, AG, and SP defined the human factor concepts at the basis of the adaptive automation. In addition they contributed to the definition of the experimental protocol. JI, GG, and RB developed the adaptive solutions used in the experiments and implemented the connection between the ATM interface and the EEG-based workload index. FB supervised and contributed to the definition of the methodologies used in the actual work and revised the entire manuscript.

### Conflict of interest statement

The authors declare that the research was conducted in the absence of any commercial or financial relationships that could be construed as a potential conflict of interest.
